# A Novel Method for Rapid Hybridization of DNA to a Solid Support

**DOI:** 10.1371/journal.pone.0070504

**Published:** 2013-08-12

**Authors:** Erik Pettersson, Afshin Ahmadian, Patrik L. Ståhl

**Affiliations:** 1 Division of Gene Technology, Science for Life Laboratory, Royal Institute of Technology, Stockholm, Sweden; 2 Cell and Molecular Biology, Karolinska Institutet, Stockholm, Sweden; Naval Research Laboratory, United States of America

## Abstract

Here we present a novel approach entitled Magnetic Forced Hybridization (MFH) that provides the means for efficient and direct hybridization of target nucleic acids to complementary probes immobilized on a glass surface in less than 15 seconds at ambient temperature. In addition, detection is carried out instantly since the beads become visible on the surface. The concept of MFH was tested for quality control of array manufacturing, and was combined with a multiplex competitive hybridization (MUCH) approach for typing of Human Papilloma Virus (HPV). Magnetic Forced Hybridization of bead-DNA constructs to a surface achieves a significant reduction in diagnostic testing time. In addition, readout of results by visual inspection of the unassisted eye eliminates the need for additional expensive instrumentation. The method uses the same set of beads throughout the whole process of manipulating and washing DNA constructs prior to detection, as in the actual detection step itself.

## Introduction

Over the past decade magnetism and magnets, both conventional and electromagnets, have found a growing field of application in the areas of biotechnology and medical technology. Combining the forces of magnetism with micro- and nanotechnology has further miniaturized the modes of application [1]. Examples of applications range from magnetoresistive-based biosensors and biochips [2], [3] to visualization of common biological events [4], [5].

Today the movement of magnetic particles in magnetic fields, most commonly beads of nanometer or micrometer sizes, has been well characterized. The force on the magnetic particle is dependent on the diameter of the particle and the strength of the magnet as well as the magnetic susceptibility of the material. In general terms, provided a certain diameter of the magnetic particle and strength of the magnet the magnetic force on the particle markedly overcomes those forces on the particle created by common diffusion [6]. Hence, the time a magnetic particle requires to move from a random point in a droplet of solution onto a surface where the droplet has been applied can be vastly shortened by applying a magnet underneath the particular surface.

In the biological sciences magnetic particles are utilized for several different purposes such as transporting, carrying or collecting biomolecules in solution. For instance magnetism has proven useful in protocols for washing [7] and DNA amplification and sequencing [8]. Attempts to improve and detect hybridization of DNA to a surface by using magnetic particles have also been undertaken using advanced sensor technologies [9], [10] and electric current [11].

Here we present a novel approach entitled Magnetic Forced Hybridization (MFH) that provides the means for efficient and direct hybridization of target nucleic acids to complementary probes immobilized on a glass surface in less than 15 seconds, without need for heating. The approach is demonstrated for the purposes of quality control of microarrays, and is combined with a method for multiplex competitive hybridization (MUCH) [12], [13] for diagnostic typing of human papilloma virus strains.

## Materials and Methods

### Ethics statement

This method development project used only synthetic and plasmid derived DNA for the HPV assay. For the quality control assay canine DNA extracted for a previous study was used.

### Samples and amplification

For the array quality control application canine DNA extracted for previous study was used. The sample was amplified targeting a 100 bp mitochondrial region as previously described [4].

The HPV plasmids (HPV 18 and 45) were normalized to 100 ng/ml by using a ND-100 spectrophotometer (NanoDrop, Wilmington, DE). The L1 region of the HPVs were PCR amplified using the general primer set GP5+/GP6+ (with the sequences GP5+ TTTGTTACTGTGGTAGATACTAC and GP6+ GAAAAATAAACTGTAAATCATATTC) where the GP6+ was biotinylated. The amplification reactions were performed as previously described [14].

### Microarray preparation

For the quality control assay 16 oligos with tiled sequences were spotted on glass slides to function as capture probes (Table S1). For the HPV assay 16 tags with different sequences were spotted on glass slides to function as capture probes (Table S1). The probes were synthesized with a 5′-poly (T) spacer of 15 thymine residues and a 5′-terminus amino link with a C6 spacer. All probes where synthesized by MWG-Biotech AG (Ebersberg, Germany). The signature tag probes were suspended at a concentration of 20 µM in 150 mM sodium phosphate, pH 8.5 and 0.06% sarkosyl solution (sarkosyl for improved spot uniformity) and were spotted using a Q-array (Genetix, New Milton, Hampshire, UK) onto CodeLink™ Activated microarray slides (7.5 cm ×2.5 cm; Surmodics, Eden Prairie, MN, USA). After printing, surface blocking was performed according to the manufacturer's instructions. The probes were printed in 16 identical arrays on the slide, and each array contained a pre-defined printing pattern. The 16 sub-arrays were separated during hybridization by a 16-pad mask (ChipClip™ Schleicher & Schuell BioScience, Keene, NH, USA).

### Preparation of on bead immobilized single-stranded DNA

A bead mixture consisting of 10 µl (100 µg) of 1 µm streptavidin-coated beads (myOne C1, Dynal, Invitrogen, Carlsbad, CA, USA) and 90 µl 2× Bind and Wash solution (10 mM Tris-HCl, 1 mM EDTA, 2 M NaCl, 1 mM Beta-Mercaptoethanol, 0.1% Tween, pH 7.5) was prepared as previously optimized [4], [5]. 30 µl of PCR product was mixed with 30 µl of the bead mixture, followed by a 5-minute incubation. In this way approximately 1 pmol product was mixed with 3 µl of 1 µm streptavidin-coated beads. Following binding of DNA the beads were washed with 1× TE buffer (10 mM Tris–HCl (pH 7.5), 1 mM EDTA). Elution of the non-biotinylated strand was carried out using 0.1 M NaOH and incubating for 4 minutes, after which the supernatant was discarded.

### Quality control assay

For the quality control assay, following removal of the non-biotinylated strand the beads were washed twice in 40 ul 1xPBS 0.2% Tween(20), and were dissolved in 30 ul 1xPBS 0.2% Tween(20).

### Multiplex competitive hybridization (MUCH) for HPV diagnostic assay

For the HPV diagnostic assay, after removal of the non-biotinylated strand the beads were washed twice in 40 µl 1× AB (Annealing buffer; 20 mM Tris-Acetate, 2 mM MgAc_2_, pH 7.6), after which the beads were resolved in 30 µl 1× AB. The immobilized single-stranded DNA was then divided into two reaction tubes (tube 1 and tube 2). Each reaction tube was subjected to a multiplex competitive hybridization (MUCH) using one out of two sets of probes. The concentration of each MUCH probe was 0.1 µM. Each MUCH probe contains a 5′-signature tag sequence that is complementary to a specific tag on the array [12] (Table S1 and S2). The MUCH probes were allowed to anneal to the immobilized template by heating the solution to 78°C for 1 min and allowing to cool down to room temperature. After removal of unbound MUCH probes the beads were washed twice in 40 µl 1× PBS 0.2% Tween(20) (Phosphate buffered saline with 0,2% Tween(20); 137 mM NaCl, 2.7 mM KCl, 10 mM Na_2_HPO_4_, 2 mM KH_2_PO_4_, pH 7.4, with 0.2% Tween(20) added), after which the beads were resolved in 30 µl 1× PBS 0.2% Tween(20). The content of the two reaction tubes were then mixed.

### Magnetic forced hybridization (MFH)

30 µl of prepared DNA-bead solution was deposited onto the microarray surface prepared with tags complementary to the signature tags on the MUCH probes. The beads were then attracted towards the surface by holding a 2-cm-diameter neodymium magnet directly underneath it for 15 seconds to allow migration of the beads to the surface and to allow for hybridization of complementary signature DNA sequences on the beads to the tags on the surface. After successful MFH the magnet was removed and the surface was washed once using 1× PBS 0,2% Tween(20).

### Detection

The results were detected by visual inspection of the surface for a pre-determined pattern. The pattern was based on how the tags were placed when generating the surface. The surface were also photographed and/or characterized by an Agilent scanner (Agilent Technologies) to determine to which locations on the surface the DNA-beads had attached through hybridization.

### Signal quantification

The array chips were scanned in an Agilent microarray scanner. The signals in the respective probe areas for HPV 18, HPV 45 and HPV 33 (unspecific signal) were obtained using Genepix Pro software (Molecular Devices, Sunnyvale, CA, USA) and integrating the average signal inside and outside the respective probe areas. From integrating the positive signal and the non-specific signal (HPV 33 probe), the signal to noise ratios could be calculated.

## Results

**Figure 1:**
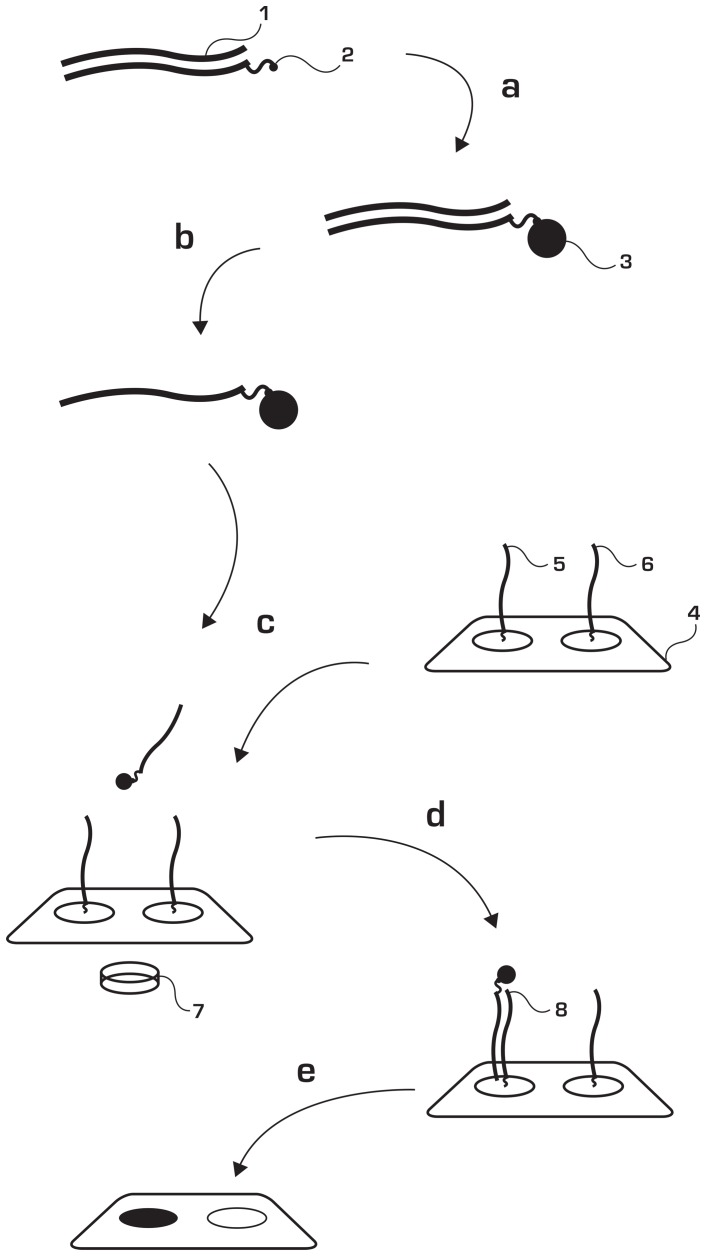
Principle of Magnetic Forced Hybridization (MFH). A double stranded PCR-product (1) carries a PCR-primer incorporated biotin affinity molecule (2). The PCR-product is bound (a) via biotin-streptavidin coupling to magnetic beads (3) and manipulated via alkali treatment (b) to become single stranded. An oligonucleotide array (4) is prepared with reverse complementary sequences (5,6) to targets of interest. The bead-product containing solution is deposited onto the detection array surface (c) and is subjected to magnetic forced hybridization using a neodymium magnet (7) under the detection surface (d). The bead-product hybridizes to its complementary oligo-tag on the array and the results are readily visible through visual inspection (e).

Figure 1 demonstrates the principle of MFH. Initially, coupling of biotinylated amplicons to streptavidin coated magnetic beads is performed in solution. Following generation of single-stranded DNA by alkali treatment the solution is added to a surface containing probes complementary to the single-stranded amplicon. Appliance of a magnetic field with a magnet introduces movement of the amplicon-carrying magnetic beads. Under the influence of the magnetic field, the beads are guided towards the surface very quickly generating a high local concentration close to the surface, which allows for the hybridization to take place. This enables a quick and specific annealing to the complementary strand on the surface within 15 seconds at ambient temperature. The detection may be carried out instantaneously since the beads become visible on the surface.

This eliminates the need for conventional hybridization, based on traditional diffusion carried out at moderate to high temperatures (above 50 degrees Celsius) and under long time-spans (1 hr –72 hrs). In addition, the method facilitates upstream template preparation since the same beads are used through the entire protocol.

**Figure 2:**
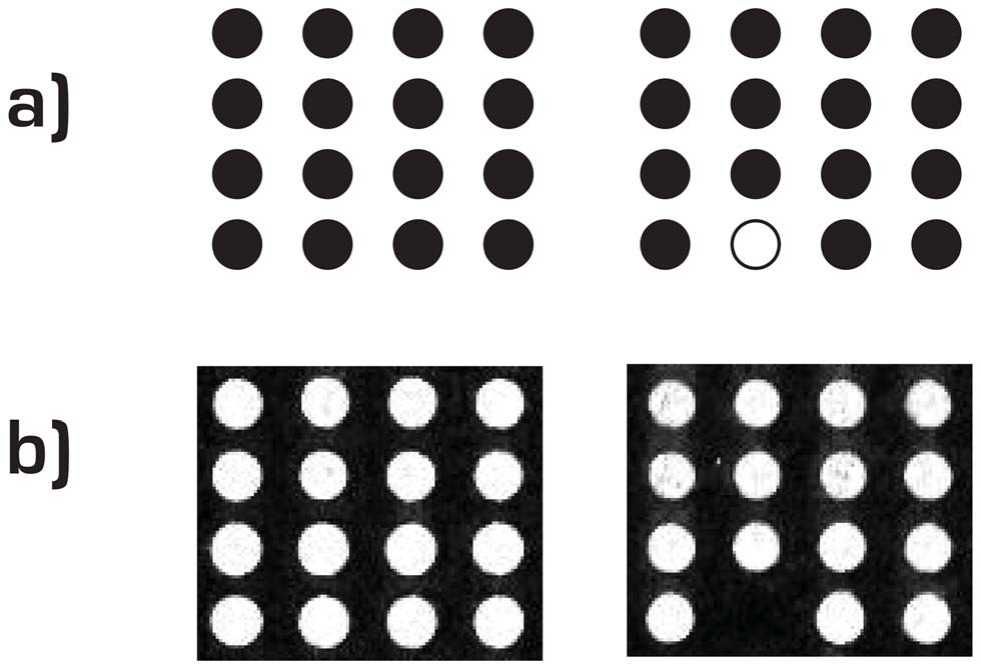
Quality control assay. a) Theoretical detection pattern for the quality control assay with complete array and defective array respectively, and b) actual results using MFH.

The most straightforward application of MFH is to hybridize an amplicon that has been made single-stranded directly to an array surface. If all probes on the array carry a common sequence, the method can be applied to test the quality of array manufacturing. The theoretical printed pattern and the results of a quality control can be seen in Figure 2 where a spot in the lower right corner of the array is missing. This direct hybridization approach has a level of discrimination that is sufficient for differing between highly unique probe sequences as is further demonstrated by the employment of universal tag sequences in the HPV diagnostic scenario described next.

**Figure 3:**
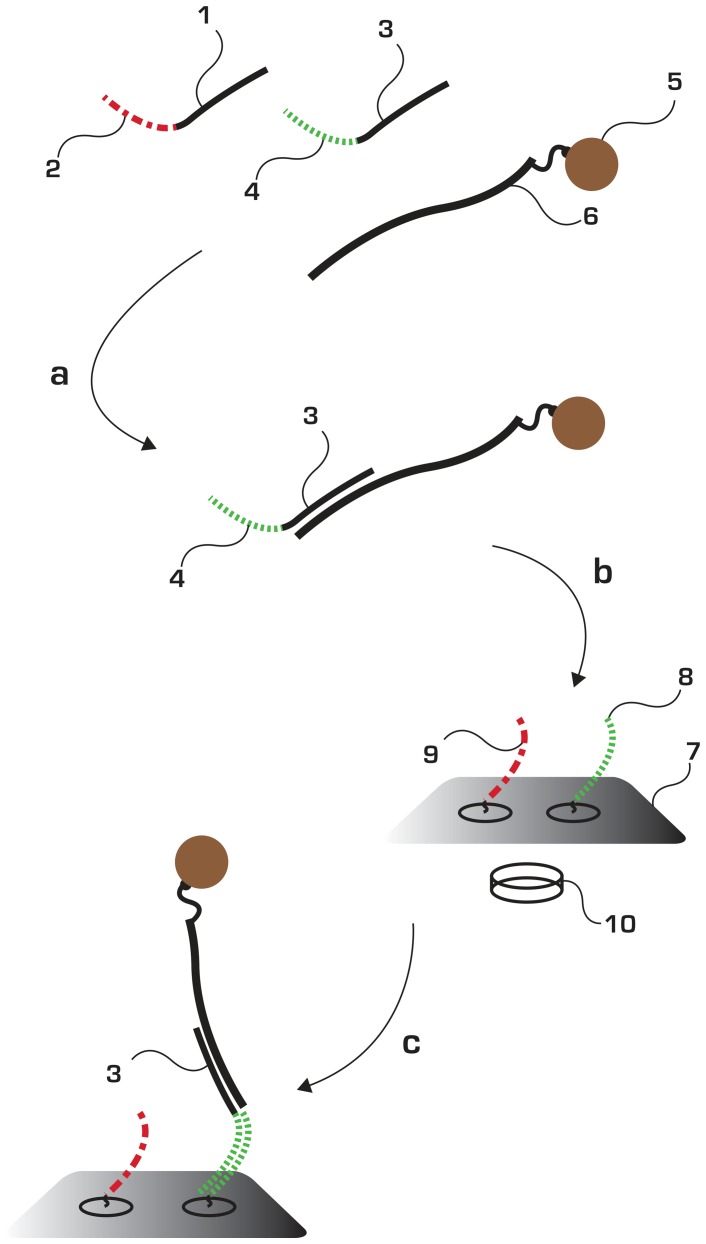
Principle of MFH in genotyping combined with Multiplex Competitive Hybridization (MUCH). MUCH-probes are designed to target all genotypes of interest. Each probe consists of two parts, one target specific region (1,3) that will hybridize and recognize a particular genotype and a corresponding address tag, designed to be orthogonal i.e. as different as possible as compared to other address tags in the pool. When (1,3) may differ at 3–5 bp positions, (2,4) are as different as possible to each other. The PCR-product (6) has been coupled to the bead (5) and made single stranded. The bead-product solution is mixed with tagged MUCH probes which are allowed to hybridize to the single-stranded amplicon following rapid heating of the sample (a). After successful MUCH, the bead-product-MUCH-tag containing solution is deposited (b) onto the detection array surface (7) and is subjected to magnetic forced hybridization using a neodymium magnet under the detection surface (10). The bead-product-MUCH-tag will hybridize via MFH to its complementary oligo-tag on the array (8). The results are readily visible through visual inspection (c) and the sample is genotyped as variant (3).

In the context of highly complex samples MFH can be combined with additional methods for genetic discrimination, such as multiplex competitive hybridization (MUCH). In this study two sets of type-specific MUCH probes that align to the L1 region of 8 HPV types (HPVs 6, 11, 16, 18, 31, 33, 40 and 45) were used [12] (Table S2). MUCH probes are allowed to competitively hybridize to the template. As shown in Figure 3, each type-specific MUCH probe has a signature tag at the 5′-end. Each of these signature sequences has a complementary sequence on the surface of the array. The magnetic field instantly guides the beads to the surface, allowing for a fast hybridization of signature tags due to a high local concentration of the target DNA.

Since there are small differences between the HPV types, the competitive MUCH approach, as an upstream protocol, is necessary to discriminate the different HPV sequences and obtain reliable results. The address tags on the MUCH-probes, however, have minimum sequence similarity and are used in the MFH that is performed at ambient temperature.

**Figure 4:**
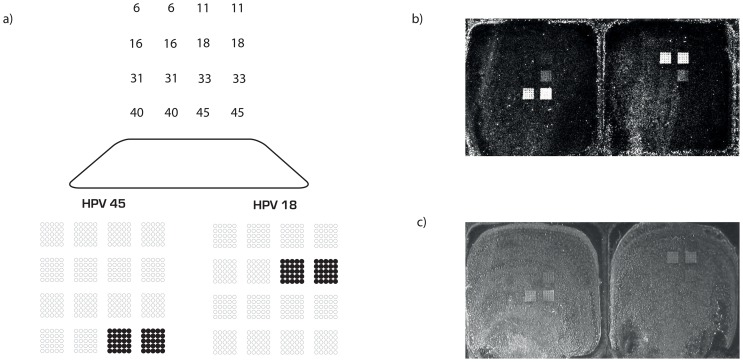
HPV diagnostic assay. a) Theoretical detection pattern for the HPV diagnostic assay for HPV 18 and HPV 45, and actual results after MFH documented with b) an array scanner and c) a digital camera. The MUCH assay requires both interrogation probes for a particular HPV species to give a positive signal, in order to give positive call for the HPV species.

In figure 4 the printed array pattern of signature tag probes can be seen together with the actual result of typing HPV 18 and 45 by combining the MUCH assay with MFH. The genotype can be interpreted by visual inspection. As originally developed [12], [13], the use of two or more sets of MUCH probes allows for a dual or multiple characterization of each HPV type, and reduces the risk of false positive readouts. In this way the HPV assay outlined here requires both probes corresponding to the given HPV type to give a positive signal in order for the assay to give a positive and true identification. The mean signal to noise ratio measured over four separate assays for the left probe corresponding to HPV 18 was 5.0, with a maximum of 7.6 and a minimum of 3.4 times over the unspecific background seen at one of the probes corresponding to HPV 33. The mean signal to noise ratio for the right probe corresponding to HPV 18 was 4.4, with a maximum of 5.6 and a minimum of 3.4. The two probes for HPV 45 generated a similarly satisfactory result with a minimum of 3.7 times signal to noise ratio over the unspecific background.

## Discussion

Hybridization of a sample molecule to a surface is a process that has traditionally required a period of anywhere from 1 hour to 72 hours. With the presented method for Magnetic Forced Hybridization (MFH) this dogma no longer holds true.

Possible applications of MFH include surface array quality control for the assessment of spot quality. Here, the most straight-forward way of using MFH is to introduce a common linker sequence into the printed array-probes. Complementary oligos attached to beads can then hybridize directly to this sequence. This provides a powerful means to quickly oversee the general printing quality and saves time compared to quality control protocols that are based on traditional hybridization. As further presented, the MFH method has applications in diagnostics and treatment where DNA testing of cell samples for presence different Human Papilloma Virus (HPV) strains is needed. Other possible applications include detection and identification of different species, or DNA testing of susceptibility mutations or disease mutations in human genomes.

The majority of the current techniques for DNA testing rely on PCR amplification prior to genotyping. This step offers improved sensitivity, allowing typing of complex and non-complex samples in combination with a range of different detection technologies. These include Sanger DNA sequencing [12], Pyrosequencing [15], and hybridization-based techniques [16], [17], [18]. The hybridization-based techniques are generally high throughput but the risk for cross-hybridizations is also high resulting in false signals.

These problems are addressed in the present work by introducing a liquid phase multiplex competitive hybridization (MUCH) of target specific probes, followed by hybridization to generic tag arrays [12], [13]. The key difference between the MUCH technique and other HPV array-based hybridization approaches is that in MUCH the discriminating (type specific) probes are mobile and have a much higher concentration compared to the target DNA allowing a reliable HPV genotyping. The approach also requires two probes to give a positive signal in order for the readout of the assay to be positive. Additionally, the assay results are highly reproducible over several experiments with satisfactory signal to noise ratios.

In conclusion, in clinical settings, the reduction in time and cost provided by the MFH method has a significant impact. In all diagnostic tests, time is a factor of great importance. With retained precision faster tests are preferred because it means decreased costs and in some cases even saving lives. Thus, a diagnostic test benefits from simple preparation procedures, inexpensive and simple equipment, cheap reagents and ease of use. Point-of-care testing to date is relying on instrumentation that is not available in all laboratories. PCR is central piece of instrumentation which can be removed entirely from the current Magnetic Forced Hybridization (MFH) protocol by the use of commercially available kits for isothermal amplification [19]. Using this or the current protocol, rapid nucleic acid hybridization for genotyping and other purposes is achievable through a minimum of processing steps and with a short processing time.

## Supporting Information

Table S1 **Surface bound tags for quality control array and HPV array.**
(DOC)

Table S2 **Signature tagged much probes.**
(DOC)
