# Supplementary material for: A Novel Method for Rapid Hybridization of DNA to a Solid Support
Source: PLoS One. 2013 Aug 12;8(8):e70504. doi: 10.1371/journal.pone.0070504 (PMC3741312; doi:10.1371/journal.pone.0070504)
Supplement: Table S1 — Surface bound tags for quality control array and HPV array. (DOC) [file pone.0070504.s001.doc]

**Table S1. Surface bound tags for QC array and HPV array**

| **Name** | **QC probe array tag sequences** |
| --- | --- |
| mt1 | GAGTTAATATGTCCTATGTAAGG |
| mt2 | GAGTTAATATGTCCTATGTAAGT |
| mt3 | GAGTTAATATGTCCTATGTAAGA |
| mt4 | GAGTTAATATGTCCTATGTAAGC |
| mt5 | TGAGTTAATATGTCCTATGTAAG |
| mt6 | TGAGTTAATATGTCCTATGTAAT |
| mt7 | TGAGTTAATATGTCCTATGTAAA |
| mt8 | TGAGTTAATATGTCCTATGTAAC |
| mt9 | GATTGAGTTAATATGTCCTATGTAA |
| mt10 | GATTGAGTTAATATGTCCTATGTAC |
| mt11 | GATTGAGTTAATATGTCCTATGTAG |
| mt12 | GATTGAGTTAATATGTCCTATGTAT |
| mt13 | GATTGAGTTAATATGTCCTATGTA |
| mt14 | GATTGAGTTAATATGTCCTATGTC |
| mt15 | GATTGAGTTAATATGTCCTATGTG |
| mt16 | GATTGAGTTAATATGTCCTATGTT |
|  |  |
| **Name** | **HPV probe array tag sequence** |
| LT3 | CCTCATGTCAACGAAGAACAGAACC |
| LT4 | ATTGAAGCCTGCCGTCGGAGACTAA |
| LT6 | TTATGGTGATCAGTCAACCACCAGG |
| LT7 | GAGACACCTTATGTTCTATACATGC |
| LT9 | GCCTTACATACATCTGTCGGTTGTA |
| LT10 | CACAAGGAGGTCAGACCAGATTGAA |
| LT12 | ACACATACGATTCTGCGAACTTCAA |
| LT13 | TTACAGGATGTGCTCAACAGACGTT |
| LT15 | CTGCACTGCTCATTAATATACTTCTGG |
| LT16 | TTCACGCACTGACTGACAGACTGCTT |
| LT18 | GCATCAGCTAACTCCTTCGTGTATT |
| LT19 | GGCGTTATCACGGTAATGATTAACAGC |
| LT21 | GCCTTATGCTCGAACTGACCATAAC |
| LT22 | CGGATATCACCACGATCAATCATAGGTAA |
| LT24 | TAGCTCTCCGCCTACAATGACGTCA |
| LT25 | AGGAACGCCTTACGTTGATTATTGA |
