# Supplementary material for: A Novel Method for Rapid Hybridization of DNA to a Solid Support
Source: PLoS One. 2013 Aug 12;8(8):e70504. doi: 10.1371/journal.pone.0070504 (PMC3741312; doi:10.1371/journal.pone.0070504)
Supplement: Table S2 — Signature tagged much probes. (DOC) [file pone.0070504.s002.doc]

**Table S2. Signature tagged MUCH probes**

| **Name** | **5’-Tag sequence** | **Type-specific sequence-3’** |
| --- | --- | --- |
| **Set 1** |  |  |
| HPV-6-1-LT3 | ggttctgttcttcgttgacatgagg | CGTAACTACATCTTCCACA |
| HPV-11-1-LT6 | cctggtggttgactgatcaccataa | TGTGTCTAAATCTGCTACA |
| HPV-16-1-LT9 | tacaaccgacagatgtatgtaaggc | CATATCTACTTCAGAAACT |
| HPV-18-1-LT12 | ttgaagttcgcagaatcgtatgtgt | TACACAGTCTCCTGTACCT |
| HPV-31-1-LT15 | ccagaagtatattaatgagcagtgcag | AATTGCAAACAGTGATACT |
| HPV-33-1-LT18 | aatacacgaaggagttagctgatgc | AGTAACTAGTGACAGTACA |
| HPV-40-1-LT21 | gttatggtcagttcgagcataaggc | CACACAGTCCCCCACACCA |
| HPV-45-1-LT24 | tgacgtcattgtaggcggagagcta | TACACAAAATCCTGTGCCA |
| **Set 2** |  |  |
| HPV-6-2-LT4 | ttagtctccgacggcaggcttcaat | TGATTATAAAGAGTACATGC |
| HPV-11-2-LT7 | gcatgtatagaacataaggtgtctc | AGATTATAAGGAATACATGC |
| HPV-16-2-LT10 | ttcaatctggtctgacctccttgtg | TACTAACTTTAAGGAGTACC |
| HPV-18-2-LT13 | aacgtctgttgagcacatcctgtaa | TGCTACCAAATTTAAGCAGT |
| HPV-31-2-LT16 | aagcagtctgtcagtcagtgcgtgaa | TAGTAATTTTAAAGAGTATT |
| HPV-33-2-LT19 | gctgttaatcattaccgtgataacgcc | GAATTTTAAAGAATATATAA |
| HPV-40-2-LT22 | ttacctatgattgatcgtggtgatatccg | TAACAGTAATTTCAAGGAAT |
| HPV-45-2-LT25 | tcaataatcaacgtaaggcgttcct | TCCTACTAAGTTTAAGCACT |
